# Quantification of Drugs in Brain and Liver Mimetic Tissue Models Using Raman Spectroscopy

**DOI:** 10.1177/00037028221139494

**Published:** 2022-11-16

**Authors:** Nathan Woodhouse, Jan Majer, Peter Marshall, Steve Hood, Ioan Notingher

**Affiliations:** 1 School of Physics and Astronomy, 6123 University of Nottingham, Nottingham, UK; 2 113460 GlaxoSmithKline, Stevenage, UK

**Keywords:** Drug delivery, drug quantification, Raman spectroscopy, microscopy

## Abstract

Quantitative analysis of drug delivery with in biological systems is an integral challenge in drug development. Analytical techniques are important for assessing both drug target delivery, target action, and drug toxicology. Using mimetic tissue models, we have investigated the efficacy of Raman spectroscopy in quantitative detection of alkyne group and deuterated drugs in rat brain and rat liver tissue models. Lasers with 671 nm and 785 nm wavelengths were assessed for their feasibility in this application due to opposing relative benefits and disadvantages. Thin tissue sections have been tested as a practical means of reducing autofluorescent background by minimizing out-of-focus tissue and therefore maximizing photobleaching rates. Alkyne-tagged drugs were quantitatively measured at 18 ± 5 μg/g drug/tissue mass ratio in rat brain and at 34 ± 6 μg/g in rat liver. Quantification calibration curves were generated for a range of concentrations from 0–500 μg/g. These results show the potential of Raman spectroscopy as a diffraction-limited spatially resolved imaging technique for assessing drug delivery in tissue applications.

## Graphical abstract

**Graphical Abstract:**
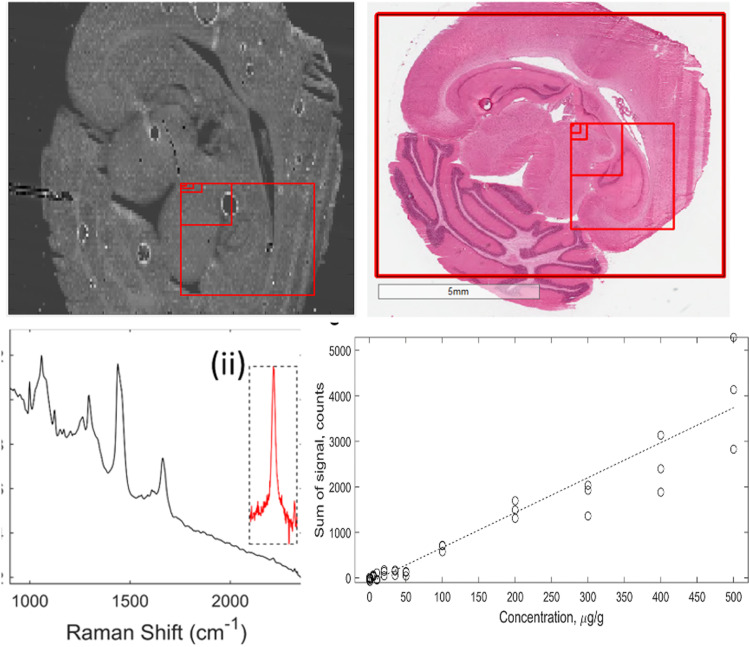

## Introduction

Confirmation of drug delivery to the site of action within a biological system is a key challenge within drug development.^1^ The ability to detect and quantify drug concentration at specific locations within biological tissue (ideally with subcellular resolution) enables both confirmation of drug delivery to desired site and assessment of potential toxicological effects elsewhere.^2^ Fluorescent protein markers can be effective tags for tracking pharmacokinetics, but the relative size of these markers compared to the molecule of interest can affect the delivery characteristics of small molecules, resulting in inaccurate analysis of the distribution of the drug in biological systems.^3^ Unlabeled or isotopically labeled techniques of drug detection enable representative drug tracking and confirmation of delivery without affecting the transport channels of the biological system. Techniques like high-performance liquid chromatography (HPLC) and mass spectrometry (MS) can detect drug molecules at very low concentrations in biological samples. HPLC is limited by the sample volume required for analysis, typically >1 μL (10^9^ μm^3^) and is unsuitable for imaging.^4^ It does, however, exhibit very high sensitivity, of order 1 ng/g in blood plasma.^4^ MS can quantitatively detect drug concentration in tissue,^5,6^ but it is also destructive and has a spatial resolution generally >100 μm^2^ (limited by the matrix crystal size sample surface).^5^

Raman spectroscopy is an optical technique that can non-destructively analyze the chemical composition of a diffraction-limited volume,^7^ which in a typical 785 nm confocal Raman microscope with 1.2 NA objective is ∼ 10 μm,^3^ by interrogating the vibrational modes in the molecules making up the volume. Coherent Raman techniques have been used extensively for detection of drug molecules in biological samples.^8–12^ Techniques like stimulated Raman spectroscopy (SRS) and coherent anti-Stokes Raman spectroscopy (CARS) can further selectively enhance the signal of chosen vibrational modes by several orders of magnitude. The spectra measured have a nonlinear relationship between the signal strength and the concentration of the drug present and exhibit nonlinear background that can be difficult to isolate from the desired drug signal^13^ in the case of CARS. These factors are not present in SRS, which does show linearity. Additional sources of noise in SRS include four-wave mixing and cross-phase modulation.^14^ These factors also make quantification difficult. Spontaneous Raman spectroscopy provides a linear response between the signal and the concentration, resulting in a linear quantification curve whose shape is unaffected by the tissue substrate the molecule of interest is being measured in. However, the limit of detection is affected by the tissue as one of the main sources of noise in Raman spectroscopy measurements in tissue is caused by the Raman and autofluorescent background from the tissue overwhelming the Raman bands of the drug.^14^ While the tissue background can generally be easily subtracted computationally,^15–17^ these methods do not reduce the shot noise added to the spectra. Tissue with high porphyrin content, like liver, is highly fluorescent under visible and near infrared illumination relative to the strength of the Raman signal. This autofluorescent background can be largely suppressed by photobleaching the sample prior to spectral measurements, but not entirely. Tissues low in porphyrins and other strongly autofluorescent molecules exhibit much lower autofluorescence under laser illumination, and as a result have lower noise contributions to the Raman signal of the drug. Thick tissue sections high in autofluorescent molecules are also at risk of thermal damage due to laser absorption during measurement.

The noise caused by the Raman bands of the tissue can be mitigated by using deuterium labels in the drug molecules of interest can, by replacing the C–H bonds with C–D bonds, shift the spectral features of the molecule into the so-called “silent region” of the Raman spectrum (∼1800–2800 cm^−1^), where there is little to no Raman signal from the tissue. The fluorescent background, however, remains for visible and near-infrared excitation wavelengths but is generally lower in magnitude than it is at lower wavenumbers. Molecules with alkyne bonds also exhibit Raman spectral features in the silent region.^11^ The reduced signal from tissue in the silent region makes isotope- and alkyne-tagged molecules easier to detect by using a region of the spectrum with reduced noise contributions from the rest of the sample. Molecules with these groups have been measured in biological samples before, using Raman techniques.^18^ The measured Raman signal is linearly proportional to the number of the relevant molecular bonds in the sampling volume, resulting in a linear relationship between the detected signal of the drug and the actual concentration of the drug within the sampled volume.^19,20^ This, combined with the lack of sample pre-processing, enables quantitative measurements of drug in tissue without requiring per-sample calibration standards.

However, eliminating the autofluorescence background from tissue is difficult, and a range of techniques have been proposed.^15–17^ A standard method of reducing this effect is using a longer wavelength of laser illumination, but the reduced quantum efficiency of charge-coupled device (CCD) detectors, and reduced scattering cross section, at longer wavelengths can diminish the benefits from the background reduction.

Here, we investigated the detection limit for deuterium- and alkyne-labeled drug molecules in mimetic tissue models of liver and brain. These are samples produced from tissue homogenate combined with the drug of interest in known quantities to produce known concentrations of the drug within tissue for assessment of the quantifiability of Raman spectroscopy for detecting these drugs. Liver is a major tissue in the metabolism of drugs and is of great interest in drug development. As a highly fluorescent tissue, it is generally difficult to measure using spontaneous Raman spectroscopy.^21^ We have chosen it for both these reasons, to assess the ability of our systems. Brain is also of interest in small-molecule drug delivery research, especially in the study of the blood–brain barrier. As a tissue that is high in fat and low in porphyrins, it is low in autofluorescence and therefore ideal for Raman measurements.

## Materials and Methods

### Instrumentation

*671 nm Raman Microscope*. The 671 nm Raman microscope was based on a continuous wave (CW) 671 nm laser (Gem 671, Laser Quantum) focused onto the sample using a 1.2 NA oil immersion objective (RiverD International, The Netherlands). The objective was also used to collect the backscattered light from the sample. This light was separated from elastically scattered laser light using a long pass dichroic filter and focused into an optical fiber coupled to a spectrometer (Shamrock 303, Oxford Instruments, UK) with a thermoelectrically cooled CCD (Newton 920, Oxford Instruments, UK) for analysis. The 671 nm illumination provides the potential benefits of increased Raman scattering and of the increased quantum efficiency of the CCD at 700–900 nm. The laser was set to illuminate the sample with 30 mW, the highest power that could reliably be used without detectable thermal damage to the samples.

*785 nm Raman Microscope*. The confocal 785 nm Raman microscope was based on a Ti:sapphire CW 785 nm laser (Model 3900s, Spectra Physics, UK) focused onto the sample using the same 1.2 NA oil immersion objective. The objective was also used to collect and collimate the backscattered light from the sample. The Raman photons were separated from elastically scattered laser light using a longpass filter and focused through a mechanical slit, to minimize transmission of out-of-focus light, and collimated into a spectrometer (Acton LS 785, Princeton Instruments) with a thermoelectrically cooled CCD (iDus 420, Oxford Instruments) for analysis. The 785 nm illumination provides the potential benefit of reduced autofluorescence from the sample relative to that from 671 nm illumination, at the cost of reduced Raman scattering and reduced CCD quantum efficiency. The laser was set to illuminate the sample with 200 mW in continuous wave mode, the highest power that had been tested beforehand that did not cause detectable thermal damage, to maximize the throughput of the instrument.

### Samples

**Figure 3.**
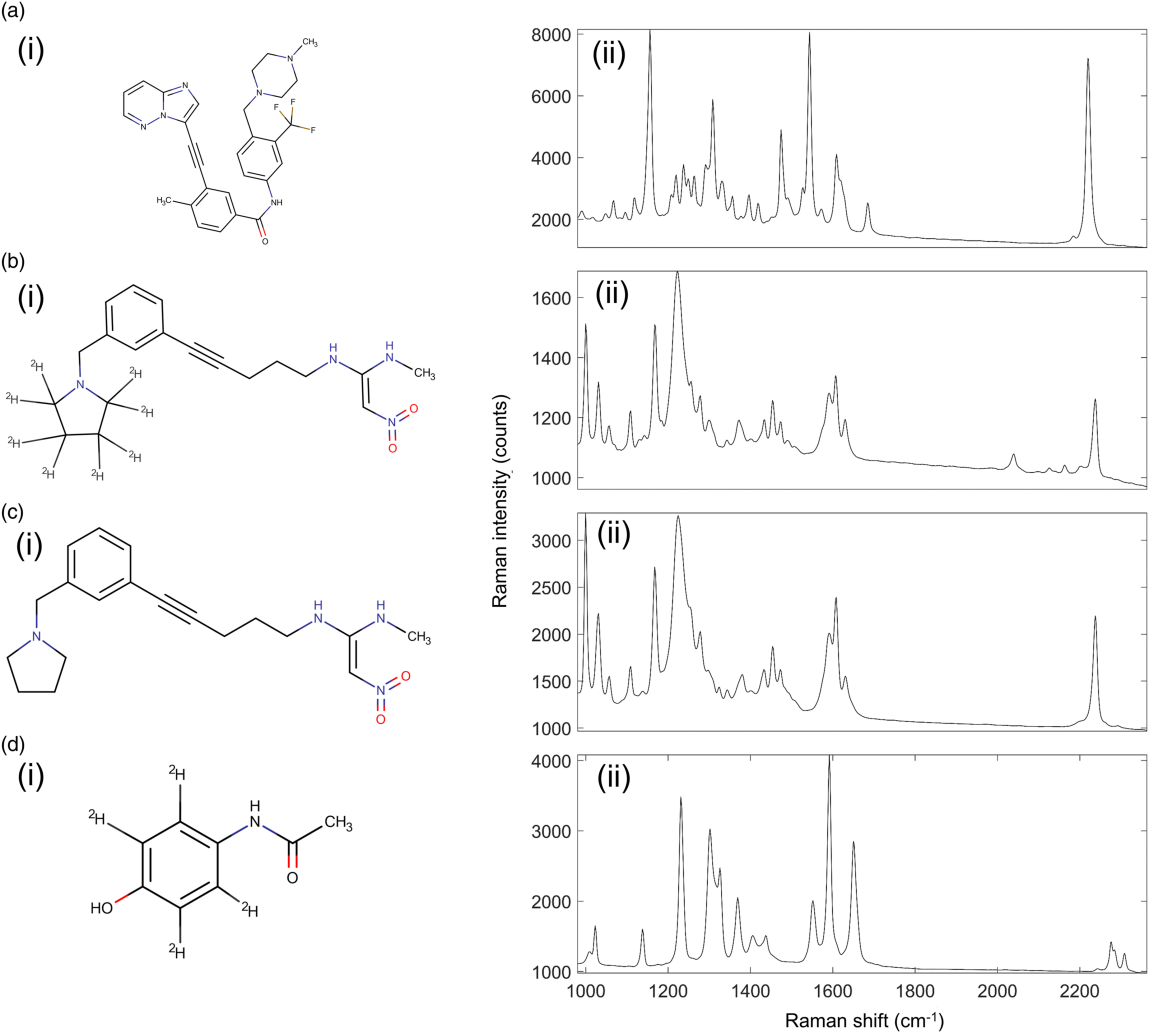
Molecular structures and 785 nm excitation Raman spectra of drugs used in this study. (a) Structure (i) and Raman (ii) spectrum of ponatinib. (b) Structure (i) and Raman (ii) spectrum of GSK4. (c) Structure (i) and Raman (ii) spectrum of GSK4x. (d) Structure (i) and Raman (ii) spectrum of acetaminophen.

Four drugs were chosen for this study and integrated into mimetic tissue models. Ponatinib is a tyrosine–kinase inhibitor used in the treatment of leukemia. GSK4X is a small molecule originally designed to inhibit activity of H1 receptors. Both molecules were chosen due to their alkyne bonds and were expected to produce a strong band in the Raman silent region. GSK4 is a deuterated form of GSK4X. This molecule has been chosen due to it containing two different molecular bonds exhibiting Raman-silent region peaks: the alkyne bond and the C–D bonds present due to its deuteration. These two drugs when compared can show the potential benefits of deuteration on the limit of detection of Raman spectroscopy. Acetaminophen is a commonly available analgesic. We investigated deuterated acetaminophen to evaluate the efficacy of Raman spectroscopy in detecting molecules with C–D bonds in tissue, in the absence of an alkyne bond. The acetaminophen was deuterated with C–D bonds replacing the C–H bonds in the benzene ring. The molecular structures and Raman spectra of the drugs are shown in Fig. 3.

### Mimetic Tissue Model Preparation

Mimetic tissue models are a reliable way of producing spatially homogenous drug–tissue combinations as an analogue of dosed tissue (ex vivo tissue obtained from animals treated with a drug). They are comprised of homogenized tissue mixed with known concentrations of the drug of interest, resulting in standardized tissue samples with known drug concentrations. They have been designed and used for quantification in mass spectrometry applications previously,^22^ where a more detailed description of their generation is available.

In short, non-dosed tissues were mechanically homogenized using steel beads and the FastPrep 24 bead homogenizer (MP Biomedicals). Homogeneous liquid tissues were aliquoted into approx. 700 mg quantities and then weighed for precise mixing of analytes. A pre-weighed mass of the drug was dissolved in a set volume of methanol to produce a drug solution with known mass concentration. For each concentration in a mimetic tissue model, a calculated volume of this solution was added to a homogenate vial. These solutions were stepped down in concentration sequentially for the lower concentration models, so the approximately the same volume of methanol would be added to each model (approx. 10 μL). Drug-spiked homogenates were then mixed using the FastPrep 24 and to homogenizing beads again. These vials of known drug/tissue mass ratio were then added sequentially to a cylindrical mold which was suspended in chilled bath of ethanol and dry ice. Allowing the liquid homogenate in the mold to freeze before adding the next homogenate prevented unwanted mixing between layers of the mimetic model. The resultant mimetic tissue model was a cylindrical block of frozen tissue homogenate, with stacked concentrations increasing along the length of the cylinder. These were then removed from the mold and mounted for vertical sectioning using the CM3050 cryostat (Leica Biosystems, Germany), which produced rectangular sections with stepped concentration increases along the long axis and constant concentration along the short axis.

For this study, mimetic tissue models were produced at 0, 0.5, 1, 5, 10, 20, 35, 50, 100, 200, 300, 400, and 500 μg/g drug/tissue mass ratio. These were made for all four of the drugs investigated, using rat brain and rat liver tissues. The models were frozen, cryosectioned at a thickness of 16 μm and thaw-mounted onto fused quartz microscope slides (UQG Optics) for Raman measurements and ITO-doped slides for matrix-assisted laser desorption–ionization mass spectrometry imaging (MALDI-MSI) measurements (Alpha Industries).

## Matrix-Assisted Laser Desorption–Ionization (MALDI) Spectrometry

The known efficacy of MALDI-MSI in detecting drugs at the concentrations used here allowed for it to be used to corroborate the drug concentrations in the mimetic tissue models.

A matrix solution was prepared by dissolution by sonification of α-cyano-4-hydroxycinnamic acid (αCHCA, Bruker Daltonics) in a 0.1% solution of trifluoric acid (TFA) in 70% EtOH to a final concentration of 7mg/mL.

An αCHCA matrix was deposited onto the sample by the automated TM-Sprayer (HTX Technologies). The automated sprayer was set to spray at 70 °C, and at matrix flow rate of 0.12 mL/min. Spraying nozzle was traversing at speed of 900 mm/min 40 mm above the sample with gas flow under pressure of 10 PSI. Spacing was set to 3 mm and the total number of passes was set to eight.

The MALDI-MSI of mimetic tissue models was performed using an Ultraflextreme II (Bruker Daltonics). The instrument was operated with flexControl software v.3.4 (Bruker Daltonics). Samples were ionized using 355 nm smartbeam-II neodymium-doped yttrium aluminum garnet (Nd:YAG) laser. Then, 400 laser shots were fired at 1 kHz frequency per raster spot (raster size = 200 × 200 µm). The analyzer was set to positive-charged reflectron mode. Fragmentation (MS/MS) analysis was performed in laser-induced forward transfer (LIFT) mode with precursor ion selector (PCIS) range set to ± 2 Da. Sampling rate was set to 0.63 GS/s. Reflector voltage was set to 2.85 kV, ion sources 1 and 2 consisted of voltages set to 7.5 kV and 6.65 kV, respectively. Lens voltage was 3.9 kV, reflectors 1 and 2 were set to 29.5 kV and 14 kV, respectively. LIFT 1 and 2 were set to 19 kV and 4.2 kV voltages, respectively. The peak detection algorithm was selected to Centroid with the TopHat baseline subtraction method. Imaging experiments were created and visualized in FlexImaging software (Bruker Daltonics, Germany).

### Raman Spectroscopy Measurements

Raman spectra of the pure drugs were measured by depositing small crystals of the drug onto fused quartz slides. To produce one Raman spectrum of each drug, several spectra acquired from approximately equally sized crystals of each drug were measured. The integration time of 0.1 s/spectrum was chosen to avoid saturation of the detector. These measurements were taken on both the 671 nm and the 785 nm Raman microscopes.

Sequentially stepped Raman maps of the control rat brain were collected with the 785 nm Raman microscope. A 2 s/spectrum integration time was chosen to facilitate large-scale mapping. Four maps of the same sample were measured: a 6.4 mm × 6.4 mm map with a 64 μm step size, a 3 mm × 3 mm map with a 32 μm step size, a 450 μm × 450 μm map with a 5 μm step size, and an 80 μm × 80 μm map with a 1 μm step size. The consecutively smaller maps were measured within the field of view of the larger maps.

Raman spectra of the mimetic tissue samples were collected with a 10 s laser dwell time per point prior to measurement for photobleaching most of the fluorescent background. After photobleaching, Raman spectra were acquired with 10 s integration for brain samples and 5 s integration for liver samples. These times were chosen to reliably maximize the signal in individual spectra without saturation of the detector. The higher autofluorescence of liver necessitated a lower integration time for this reason. For longer overall acquisition times, repeat measurements of Raman spectra at the same positions in the sample were recorded and then averaged. Due to the high well depth and low readout noise of the CCDs operating in single-track mode, the noise added into the measurements by summing sequential spectra was negligible relative to the shot noise from the background autofluorescence when the integration times are set to maximize use of the dynamic range of the CCDs. To record the fingerprint region of the tissue and the silent region peaks of the drugs, the spectral range chosen was 900–2400 cm^−1^ (avoiding quartz Raman bands below 900 cm^−1^). The spectral resolution of the systems in this range was 3 cm^−1^. To minimize potential tissue degradation during measurement, the 785 nm instrument was fitted with a cooled stage maintained at 8 °C during the long limit of detection measurements. This was found to be the lowest temperature that the samples could be kept at without risking water condensation build-up around the optics.

### Detection Limit Measurements

For measuring the limit of detection of drugs in tissue, three randomly sampled locations in each concentration of each mimetic tissue model were measured to assess the detection limit of each mimetic tissue model and the variability in the samples. The total acquisition time for each location depended on the concentration of drug in the mimetic tissue model. At 100, 200, 300, 400, 500 μg/g, the measurement times were 60 min, 30 min, 15 min, 10 min, and 5 mins, respectively. At all concentrations below 100 μg/g, the acquisition time used was 120 min.

### Spectral Analysis

#### Spectral Subtraction and Normalization

For the large field of view (FOV) Raman map of rat brain tissue, the images presented were generated using the area under the 1450 cm^−1^ Raman peak corresponding to CH_2_ bending.

To account for variance in the thickness of the tissue samples in the mimetic tissue models, spectra were normalized by scaling the silent region 1800 cm^−1^ to 2150 cm^−1^ of the drugged samples to that of a reference 0 μg/g measurement. This region is invariant between samples other than absolute magnitude, so acts as a reference point to scale Raman signal with. The 0 μg/g spectrum was then subtracted from the spectra, removing the autofluorescent background and most of the reproducible interference pattern from the silent region. This interference pattern, being an artifact of the CCD, was almost identical in each measurement and as such can generally be eliminated through background subtraction.

### Limit of Detection

The limit of detection is the lowest drug concentration that can be measured. The signal measured from a region of tissue with a lower drug concentration is not statistically discriminable from the signal measured from a region of tissue with 0 drug concentration and is therefore indistinguishable from undosed tissue.

The limit of detection of the system is dependent on the drug of interest, the tissue of interest, and the integration time used. This is because the limit of detection^21^ is reached when the standard deviation in the measured signal from a non-drugged (blank) mimetic tissue model, σ_blank_, and the standard deviation in the measured signal from a drugged mimetic tissue model, σ_lim_, reaches an overlap described by

(1) $$\mu_{blank}+1.645\sigma_{blank}=\mu_{\lim}-1.645\sigma_{\lim}$$

where μ_lim_ and μ_blank_ are the average signals measured from the detection-limited mimetic tissue model and the non-drugged mimetic tissue model, respectively. The limit of detection can be approximated for a certain integration time using the mimetic tissue models described here by finding the lowest concentration in the mimetic tissue model where this relation (Eq. 1) holds true. As the signal measured is proportional to the concentration of the drug, this limit can be interpolated between the lowest concentration measured and the highest concentration not measured to approximate the actual detection limit.

## Results and Discussion

### Large Field of View Raman Mapping of Thin Tissue Section Modeling

**Figure 1.**
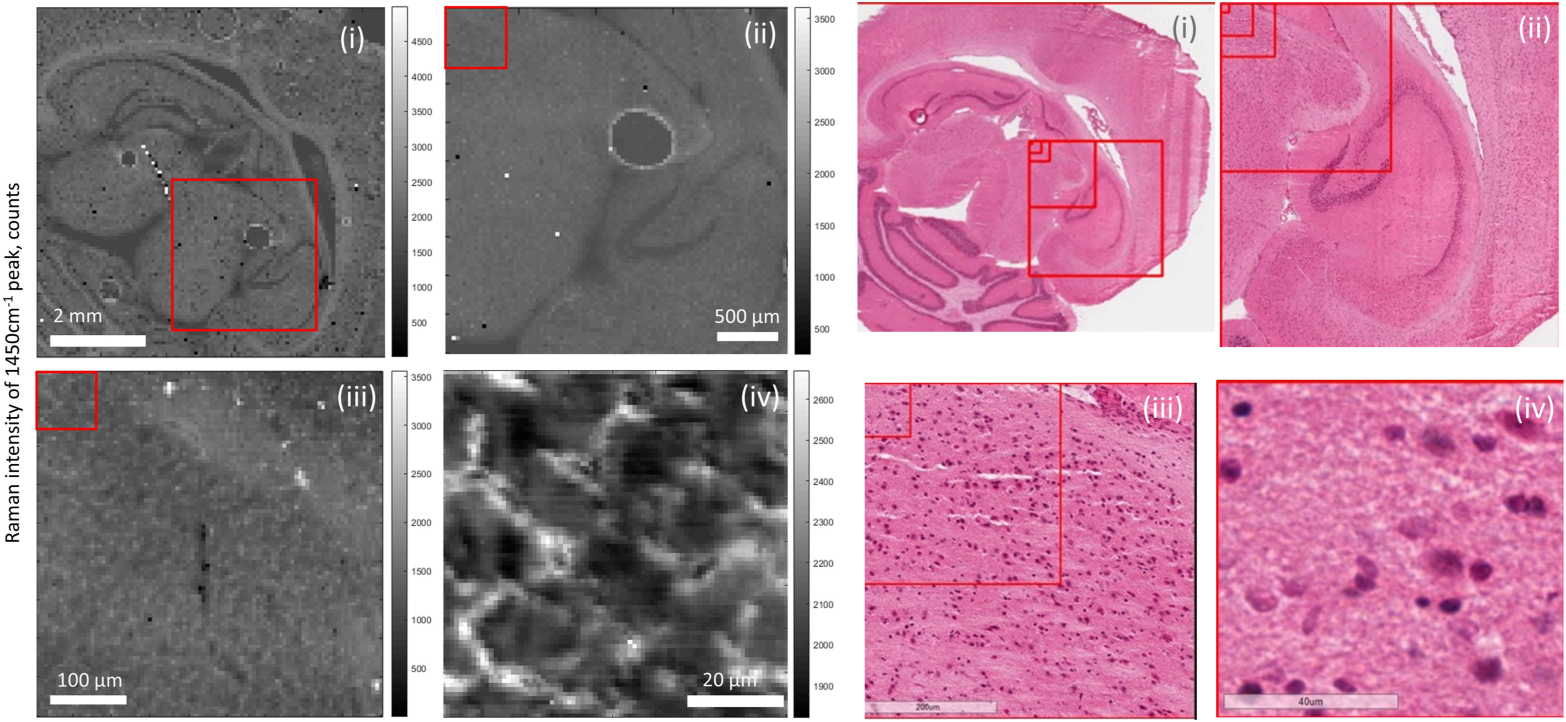
Series of 785 nm excitation Raman maps of 1450 cm^−1^ peak of rat brain section. Adjacent histology sections are included for comparison. (i) 6.4 mm x 6.4 mm map with a 64 μm step size, red box indicates FOV of (ii). (ii) a 3 mm x 3 mm map with a 32 μm step size, red box indicates FOV of (iii). (iii) a 450 μm x 450 μm map with a 5 μm step size, red box indicates FOV of (iv). (iv) 80 μm x 80 μm map with a 1 μm step size. Scale bars inset.

Due to the long measurement times associated with Raman spectroscopy, full field of view (FOV) scanning of entire samples is impractical. The diffraction-limited spatial resolution enables single-point measurements of < 1 μm regions within large tissue samples. A consecutive series of Raman maps of the same sample at different length scales are shown in Fig. 1. At the highest FOV, organ structures of rat brain are discernible, including the neocortex, corpus callosum, and cerebellum. At the lowest FOV, the structures of individual cells are visible.

Mitigation of tissue autofluorescence background and laser wavelength selection Tissue background fluorescence was minimized by sectioning the tissue at approximately the same thickness as the depth of focus of the microscopes (∼16 μm). This enabled the system to have the maximum Raman spectral sensitivity while minimizing the presence of out-of-focus tissue that contributes high autofluorescence when attempting bulk tissue measurements. Low overall laser attenuation in the sample due to its low thickness also minimized the risk of thermal damage during photobleaching. Due to the long measurement times and high laser powers used in this study, photobleaching occurred naturally by the illumination laser.

Near-infrared lasers (e.g., 785 nm wavelength) are commonly used in Raman spectroscopy for biological materials to a reduced fluorescent background. However, the quantum efficiency of CCD detectors falls off rapidly above around 900 nm, which corresponds to around 1620 cm^−1^ Raman shift for 785 nm excitation. As a result, the Raman signal intensity in the silent region (1800–2800 cm^−1^) is reduced relative to the fingerprint region (800–1800 cm^−1^). With a 785 nm system the quantum efficiency at 2220 cm^−1^, the location of one of the drug peaks detected in this study, is around 60%, relative to >80% for the fingerprint region. Using a 671 nm laser for excitation solves this issue by shifting all the measured wavelengths lower, into the spectral range where the CCD is most efficient. The resultant quantum efficiency of the system at 2220 cm^−1^ is > 90%, a ∼ 50% relative improvement in signal sensitivity over a 785 nm system. This combined with the higher scattering cross section of 671 nm results in a theoretical 2.8-fold increase in detected Raman photons relative to 785 nm, for a Raman band at 2200 cm^−1^. Thus, to improve the detection limit of drugs in tissue, the increased autofluorescent background must not be higher by the same factor, or the signal-to-noise ratio (SNR) of the system will be inferior. To compare the two wavelengths, the ponatinib mimetic tissue models of both brain and liver at 0, 100, and 500 μg/g were measured using both the 671 nm and 785 nm Raman instruments. Ponatinib drug was chosen due to it having the strongest measured signal of the mimetic tissue models produced. The acquisition times were the same for both instruments (5 min for each point, but the laser powers were different (listed in the instrumentation section) due to limitations of possible laser damage to the samples.

**Figure 2.**
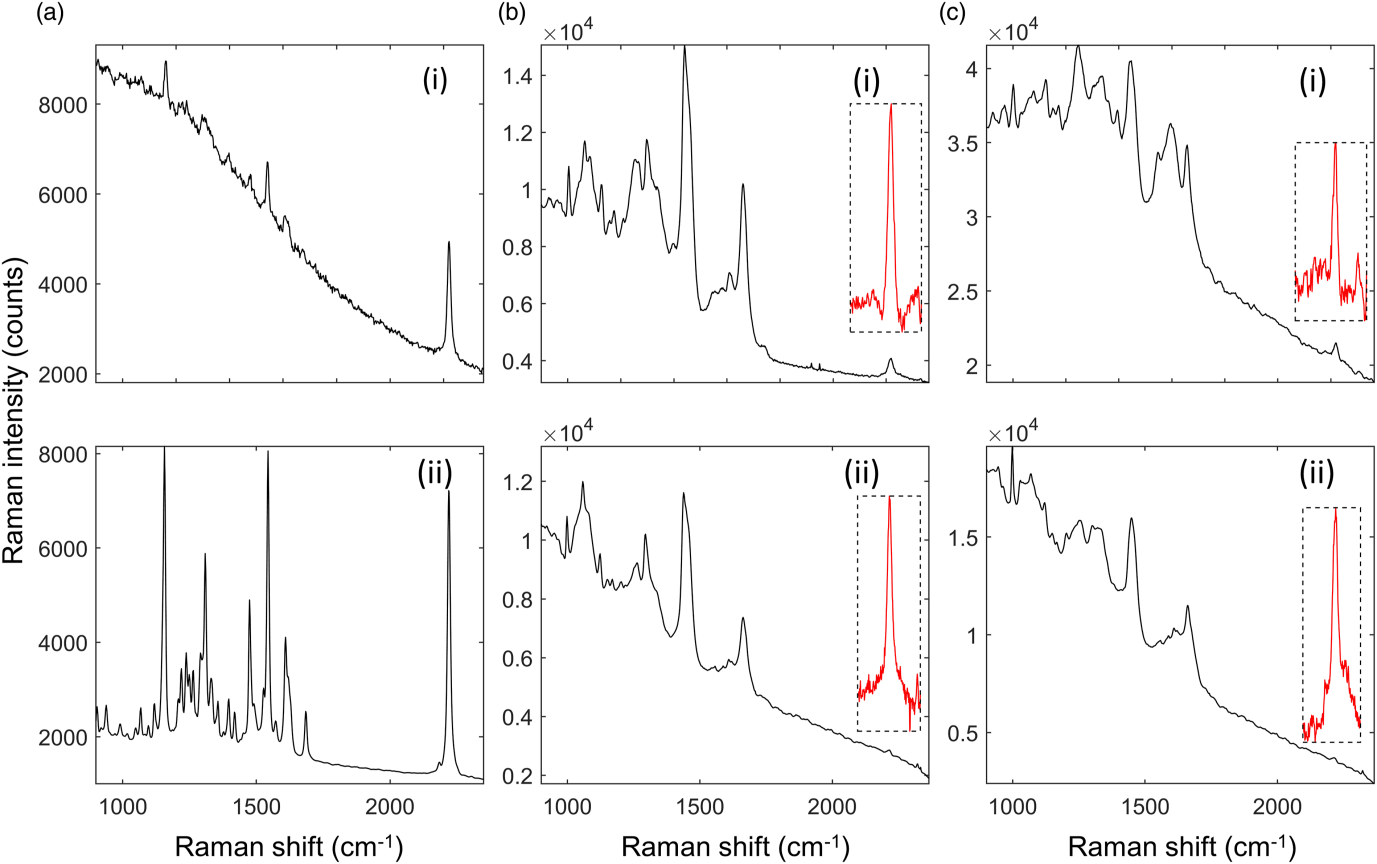
Comparison of Raman spectra of ponatinib and mimetic tissue models (brain and liver) measured using 671 nm and 785 nm excitation wavelengths. (a) Raman spectra of crystalline ponatinib: (i) 671 nm and (ii) 785 nm (integration time: 0.1 s). (b) Raman spectrum of 500 μg/g ponatinib/brain mimetic tissue model measured using (i) 671 nm, (ii) 785 nm (integration time: averages of 30 spectra each, 10 s/spectrum). Inset in box, 2220 cm^−1^ peak after subtraction of Raman spectrum of pure tissue, magnified to show noise levels. (c) Raman spectrum of 500 μg/g ponatinib/liver mimetic tissue model measured using (i) 671 nm, (ii) 785 nm (integration time: average 60 spectra at 5 s/spectrum). (Insert) 2220 cm^−1^ peak after pure tissue subtraction, magnified to show noise levels.

Figure 2 compares Raman spectra measured using 671 nm and 785 nm excitation wavelengths of ponatinib in brain and liver tissue. The range of the spectrum below 1800 cm^−1^ is the fingerprint region of the Raman spectrum. The peaks in this range can be attributed to the pyrimidine group, the phenyl rings, and the trifluoromethyl group in the molecule, along with an alkyne peak at ∼1600 cm^−1^. While the 2220 cm^−1^ peak of ponatinib is more intense than the bands in the fingerprint region using the 671 nm laser (Fig. 2a(i)), compared to those using the 785 nm laser (Fig. 2a(ii)), the 671 nm also induces a higher autofluorescence.

This auto-fluorescence background increases the noise of the measurements in the mimetic tissue models on top of the noise from the tissue itself. For tissue (Fig. 2b), the visible bands in these spectra include the phenylalanine peak at 1001 cm^−1^, amide III bands in the 1200–1300 cm^−1^ range, the CH_2_ bend in proteins and lipids at 1450 cm^−1^, and the amide I peak at 1660 cm^−1^. Different concentrations of these biomolecules cause the difference in the strength of these peaks between brain and liver. These Raman peaks present in the tissue overlap with the peaks in the drug spectra, making discrimination difficult. The inset plots of background-subtracted spectra in Figs. 2b and 2c are magnified to show the strength of the 2220 cm^−1^ Raman peaks relative to the noise in the spectra. The noise, which is primarily shot noise from the autofluorescent background of the tissue, is proportional to the square root of the total signal measured. As a result, a higher background level increases the noise in the measured spectra. Figure 2 shows that tissue background in the silent region from brain is slightly higher using 671 nm compared to 785 nm, but much higher, by a factor of ∼4, for liver.

The SNR was calculated here as the area under the 2220 cm^−1^ peak (signal) divided by the standard deviation of the background-subtracted spectrum in a flat part of the silent region (noise). When the background was subtracted, this region is flat and mostly constant across a given tissue type. For the ponatinib/rat brain mimetic tissue model at 500 μg/g, the SNR was ∼650 for the 671 nm system and 1100 for the 785 nm system. Therefore, 785 nm could produce ∼1.7-fold higher SNR and a had a detection limit ∼0.6-fold smaller than that of 671 nm. For the ponatinib/rat liver mimetic tissue model at 500 μg/g, the SNR was ∼260 for the 671 nm system and ∼950 for the 785 nm system. Therefore, 785 nm could produce ∼3.7 × the SNR and a detection limit ∼0.27 × that of 671 nm. These results show that, especially for highly fluorescent tissues like liver, 785 nm was more effective in detecting the drug based on Raman bands in the silent region, despite the fact that the quantum efficiency of the detector in this spectral region was lower. The same was true for less fluorescent tissues like brain, but the difference was less extreme and other factors may be considered before choosing which wavelength to use. As a result of these tests, full scale measurements on the mimetic tissue models were only performed on using the 785 nm system.

### Raman Spectra of Drugs

The chemical structures and respective Raman spectra are shown in Fig. 3. Ponatinib exhibited a strong Raman peak in the silent region, at 2220 cm^−1^ assigned to the alkyne bond present in the molecule. This peak also had a weak shoulder on the lower wavenumber side at around 2180 cm^−1^, also a result of the alkyne bond, which is visible in Fig. 3a(i). GSK4 exhibited several Raman peaks in the silent region, at 2031 cm^–1^, 2118 cm^–1^, 2159 cm^–1^, and 2236 cm^−1^, as shown in Fig. 3b(ii). The 2236 cm^−1^ peak is a feature from the alkyne bond in the molecule, while the others are a feature of the C–D bonds in the molecule. GSK4x exhibited a single Raman peak in the silent region, at 2236 cm^−1^, as shown in Fig. 3c(ii). This peak, as in GSK4, was attributed to the alkyne bond in the molecule. Deuterated acetaminophen exhibited 2 weak Raman peaks in the silent region, at 2276 cm^−1^ and 2309 cm^−1^. These were both attributed to the C–D bonds present in the molecule.

### Quantification of Drugs in Biomimetic Tissue Models

**Figure 4.**
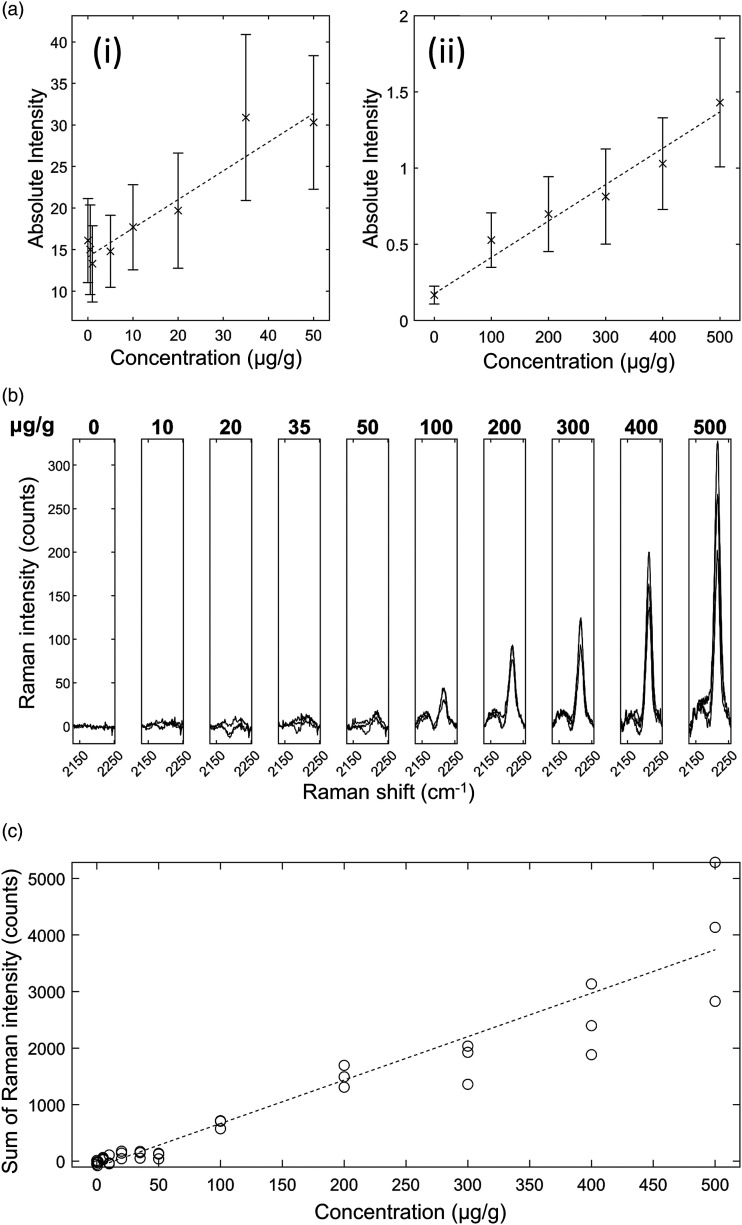
Ponatinib in rat brain mimetic tissue model. (a) Weighted regression fit (i) of MALDI intensity of 183.56 Da mass peak for 0–50 μg/g mimetic tissue model (dotted line), mean measured signal at given concentration and standard deviation (crosses and bars). (a) Weighted regression fit (ii) of MALDI intensity of 183.56 Da mass peak for 0–500 μg/g mimetic tissue model. (b) Integration time-normalized 2220 cm^−1^ peak in mimetic tissue models. Three randomly sampled locations of each mimetic tissue model were measured for each concentration. (c) Linear regression fit of sum of signal under 2220 cm^−1^ peak at given concentration (dotted line), measured signal under peak at given concentration for each sample (circles).

*Ponatinib in Brain Mimetic Tissue Model.* The Raman spectra for the ponatinib in brain mimetic model are shown in Fig. 4. The 2220 cm^−1^ peak, after tissue spectrum subtraction, was visible in the spectra of drugged mimetic tissue models at higher concentrations as in Fig. 4b. After baseline subtraction, the Raman band was clearly visible as shown in Fig. 4b. Because the spectra were normalized to total integration time and to the pure reference tissue mimetic model spectrum of each sample, the strength of the peaks can be compared directly for developing quantitative models. The lowest concentration with a visible Raman peak at 2220 cm^−1^ measured was 35 μg/g, and the lowest concentration with a significant measured signal (area under peak), as defined in Eq. 1, was 20 μg/g. The quantification curve had a gradient of 7.68 counts/(μg/g), with *r* = 0.95. The limit of detection was 18 ± 5 μg/g.

**Figure 5.**
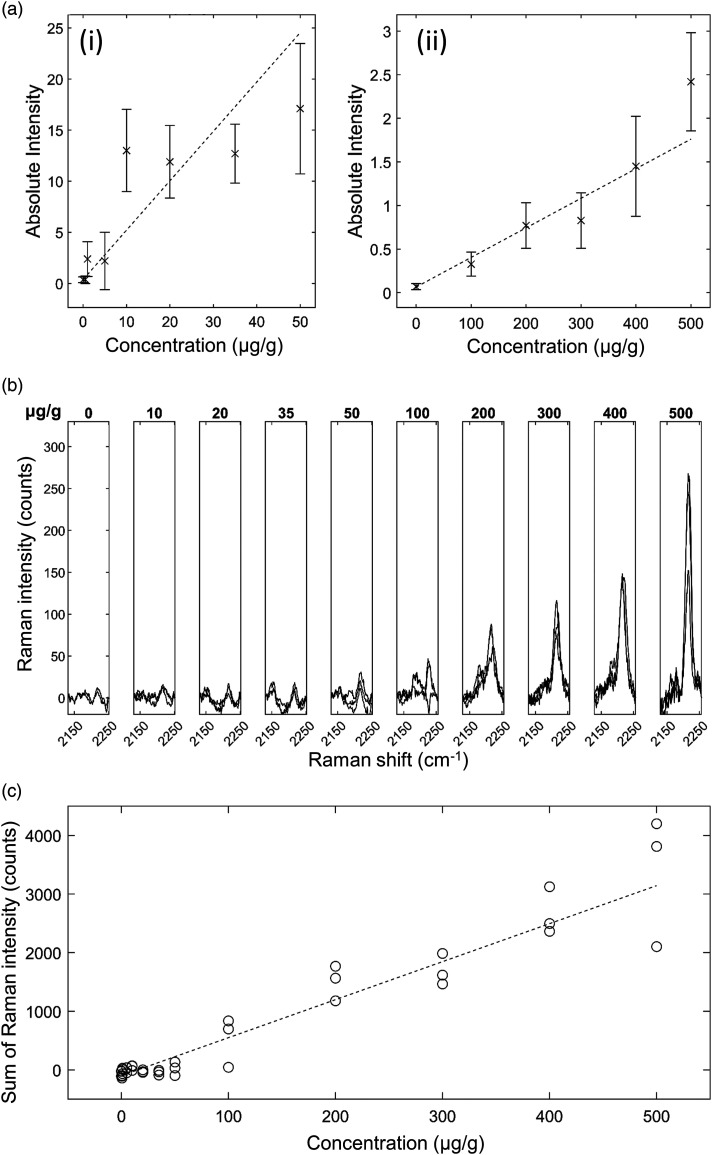
Ponatinib in rat liver mimetic tissue model. (a) Weighted regression fit (i) of MALDI intensity of 183.56 Da mass peak for 0–50 μg/g mimetic tissue model (dotted line), mean measured signal at given concentration and standard deviation (crosses and bars). (a) Weighted regression fit (ii) of MALDI intensity of 183.56 Da mass peak for 0–500 μg/g mimetic tissue model. (b) Integration time-normalized 2220 cm^−1^ peak in mimetic tissue models. Three randomly sampled locations of each mimetic tissue model were measured for each concentration. (c) Linear regression fit of sum of signal under 2220 cm^−1^ peak at given concentration (dotted line), measured signal under peak at given concentration for each sample (circles).

*Ponatinib in Liver Mimetic Tissue Model*. The results for ponatinib in liver are shown in Fig. 5. The 2220 cm^−1^ peak and 2180 cm^−1^ shoulder were visible after baseline subtraction in the spectra of drugged mimetic tissue models at higher concentrations (Fig. 5b). The bands were sufficiently strong at 500 μg/g concentration to be visible above in the non-baseline subtracted spectrum. Because the spectra were normalized to total integration time and to the pure reference tissue mimetic model spectrum of each sample, the strength of the peaks can be compared directly to make quantitative predictions. The minimum concentration with a visible Raman peak at 2220 cm^−1^ measured was 50 μg/g, and the minimum concentration with a significant measured signal was 35 μg/g at the integration time used. The quantification curve had a gradient of 6.48 counts/(μg/g), with *r* = 0.95. The limit of detection was 34±6 μg/g.

**Figure 6.**
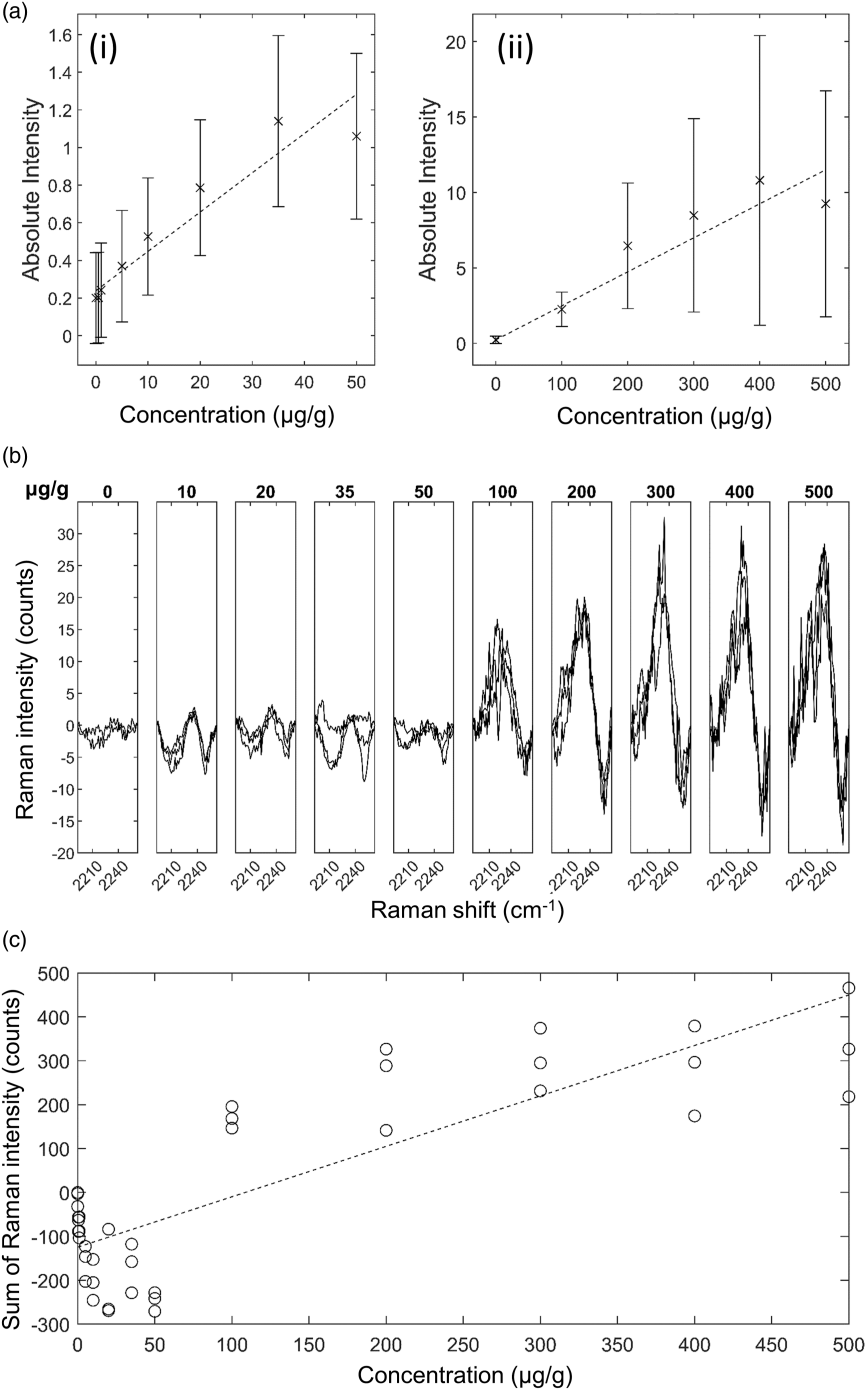
GSK4 in rat brain mimetic tissue model. (a) Weighted regression fit (i) of MALDI intensity of 250.6 Da mass peak for 0–50 μg/g mimetic tissue model (dotted line), mean measured signal at given concentration and standard deviation (crosses and bars). (a) Weighted regression fit (ii) of MALDI intensity of 250.6 Da mass peak for 0–500 μg/g mimetic tissue model. (b) Integration time-normalized 2236 cm^−1^ peak in mimetic tissue models. Three randomly sampled locations of each mimetic tissue model were measured for each concentration. (c) Linear regression fit of sum of signal under 2236 cm^−1^ peak at given concentration (dotted line), measured signal under peak at given concentration for each sample (circles).

*GSK4 in Brain Mimetic Tissue Model.* The results for GSK4 in brain are shown in Fig. 6. After baseline subtraction, the 2236 cm^−1^ peak was visible as shown in Fig. 6b. The lowest concentration with a visible Raman peak at 2236 cm^−1^ measured was 100 μg/g, and the lowest concentration with a significant measured signal (area under peak) was 100 μg/g. The quantification curve had a gradient of 1.14 counts/(μg/g), with *r* = 0.82. The limit of detection was 80 ± 14 μg/g. The other silent region peaks corresponding to C–D bonds were not detected at the concentrations and measurement times used in this study.

**Figure 7.**
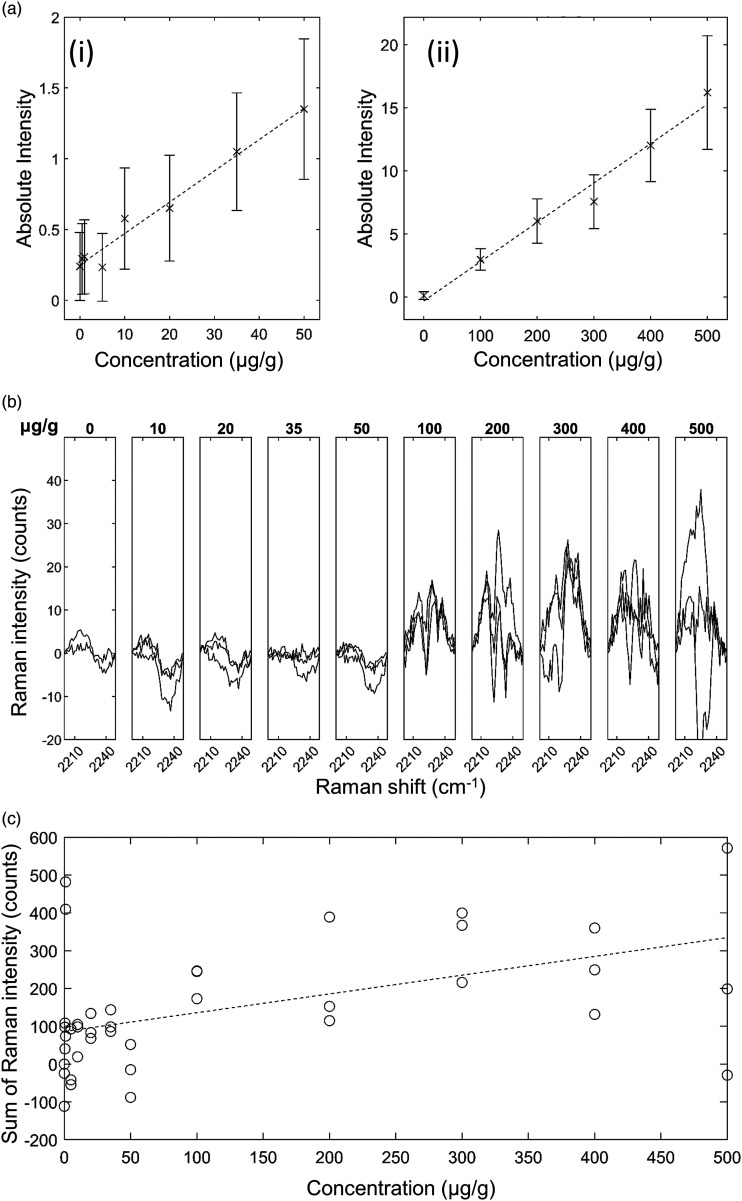
GSK4 in rat liver mimetic tissue model. (a) Weighted regression fit (i) of MALDI intensity of 250.6 Da mass peak for 0–50 μg/g mimetic tissue model (dotted line), mean measured signal at given concentration and standard deviation (crosses and bars). (a) Weighted regression fit (ii) of MALDI intensity of 250.6 Da mass peak for 0–500 μg/g mimetic tissue model. (b) Integration time-normalized 2236 cm^−1^ peak in mimetic tissue models. Three randomly sampled locations of each mimetic tissue model were measured for each concentration. (c) Linear regression fit of sum of signal under 2236 cm^−1^ peak at given concentration (dotted line), measured signal under peak at given concentration for each sample (circles).

*GSK4 in Liver Mimetic Tissue Model. *The results for GSK4 in liver are shown in Fig. 7. The 2236 cm^−1^ peak, after tissue spectrum subtraction, was visible in the spectra of drugged mimetic tissue models at higher concentrations as in Fig. 7b. High levels of background variation were visible in these spectra, along with high signal variation in the 500 μg/g measurements. The other bands assigned to C–D in the silent region were not detected. Time-normalization of the spectra enables direct peak strength comparison between the measurements of different drug concentrations. The lowest concentration with a visible Raman peak at 2236 cm^−1^ measured was 100 μg/g, and the lowest concentration with a significant measured signal was 100 μg/g. The quantification curve had a gradient of 0.8 counts/(μg/g), with *r* = 0.5. The limit of detection was 170±20 μg/g.

*GSK4x in Brain Mimetic Tissue Model*. The results for GSK4x in brain are shown in Figure S1 (Supplemental Material). The 2236 cm^−1^ peak, after tissue spectrum subtraction, was visible in the spectra of drugged mimetic tissue models at higher concentrations as in Figure S1b. High background variation was visible in these spectra, especially in the 35 μg/g measurements. The lowest concentration with a visible Raman peak at 2236 cm^−1^ measured was 100 μg/g, and the lowest concentration with a significant measured signal (area under peak) was 300 μg/g. The quantification curve had a gradient of 1.58 Counts/(μg/g), with *r* = 0.76. The limit of detection was 270 ± 30 μg/g.

*GSK4x in Liver Mimetic Tissue Model*. The results for GSK4x in tissue are shown in Figure S2 (Supplemental Material). GSK4x exhibited a single Raman peak in the silent region, at 2236 cm^−1^. The 2236 cm^−1^ peak, after tissue spectrum subtraction, was visible in the spectra of drugged mimetic tissue models at higher concentrations as in Figure S2b. Because the spectra have been normalized to total integration time and to the pure reference tissue mimetic model spectrum of each sample, the strength of the peaks can be compared directly to make quantitative predictions. The lowest concentration with a visible Raman peak at 2236 cm^−1^ measured was 200 μg/g, and the lowest concentration with a significant measured signal (area under peak) was 300 μg/g. The quantification curve had a gradient of 0.42 Counts/(μg/g), with *r* = 0.7. The limit of detection was 2900 ± 300 μg/g. This limit of detection is much higher than that of the GSK4x brain mimetic model despite the peak being visible in the same range. The interference fringes in the spectra were sufficiently strong in these measurements to cause significant overlap with the peak such that, while the 2236 cm^−1^ peak could be seen at 400 μg/g, it was difficult to discriminate from the background fringes without looking at the adjacent concentration spectra. This resulted in a 0-signal measured at this concentration that impaired the quantification curve fitting. These interference fringes also resulted in a high noise floor that could not be reliably subtracted.

Drug spectral features were found in all mimetic tissue models except for the models measuring deuterated acetaminophen. However, the deuterated acetaminophen models could not be verified with MALDI imaging (considered standard of reference), which also could not detect the drug.

With the combined use of thin tissue sections and high laser power, the autofluorescence emission of tissue was mitigated, enabling acquisition of Raman spectra of both brain and liver tissue and allow detection of drugs in the range 18–300 μg/g. The linearity of the Raman response to the drug concentration agreed with the response of the MALDI measurements used to verify the accuracy of the mimetic tissue models. The sample geometry also lends itself to high spatial resolution measurements, as the significant out-of-focus tissue was removed. As measurements are non-destructive, it is also potentially useful for multivariate imaging, where consecutive sections of tissue can be measured with different imaging modalities.^22,23^ The detected range is clinically relevant for bulk plasma detection of some drugs, which can be as high as 1000 μg/g.^24^

Using 785 nm laser for excitation in Raman spectroscopy led to a higher SNR and therefore lower detection limit for detecting drug in the tissues compared to 671 nm laser. This was due to the reduced autofluorescent background and higher potential laser illumination power. The difference, however, was less extreme than expected, less than one order of magnitude. This result means that many factors in future experiments could affect which wavelength to use, including the specific tissue and drugs being measured. Tissues with very low fluorescence emission, or drugs with relevant Raman peaks in the high wavenumber region (>2800 cm^−1^), or high in the silent region may demonstrate better results with 671 nm. Instruments with detectors that have been designed to reduce the oscillating background caused by interference could also make the lower laser wavelengths the better choice. Conversely, for tissues exhibiting very high autofluorescence or drugs with discriminable peaks at lower wavenumbers, higher laser wavelengths than 785 nm may improve the detection limits of spontaneous Raman spectroscopy.

Ponatinib in brain was the drug/tissue mimetic model combination measured with the lowest limit of detection, due to ponatinib having the strongest silent region peak and brain having the lowest noise contribution, of the drugs and tissues measured in this study. The higher limit of detection in the ponatinib/liver mimetic tissue model relative to that of the brain mimetic tissue model was due to the increased background noise from the liver, as well as the increased background causing the interference pattern in the CCD to become relatively stronger. This pattern overlapped with the peak of interest and reduced certainty in what was or was not drug signal at lower concentrations.

For the GSK4/brain mimetic tissue model, the limit of detection relative to that of the ponatinib mimetic tissue model was higher, which was likely primarily due to the significantly reduced drug signal in the mimetic tissue models, about a 10× decrease in signal relative to background for a given concentration. The only measurable peak in the silent region was that from the alkyne group, with the deuteration peaks not visible in the mimetic tissue model spectra. In the GSK4/liver mimetic tissue model, the quantification curve had little correlation to actual concentration, which was exacerbated by high relative noise levels in the high concentration models. The poor correlation could be due to metabolism of the drug in the tissue, as the tissues have not been fixed prior to measurement. Quantitative measurement of this drug/tissue combination would require longer measurement times at these concentrations to generate a reproducible quantification curve.

For the GSK4X mimetic tissue models, the limits of detection were much higher than that of the GSK4 mimetic tissue model despite the peak being visible in the same range. The noise levels in these models were less consistent, leading to great increases in background as can be seen in Fig. 6b, where one of the spectra at 20 μg/g were much higher than the drug signal should have been.

The comparison of GSK4 and GSK4X shows that deuteration is a much weaker label for spontaneous Raman spectroscopy measurements than alkyne groups. Combined with the results from deuterated acetaminophen, none of the Raman peaks assignable to C–D bonds were detected in this study. The alkyne group band at ∼2220 cm^−1^ was reliably measurable from all mimetic tissue models that contained an alkyne group.

The variance in the drug concentration of the mimetic tissue models at 200 μm scale was observed in the MALDI imaging results. The samples were not investigated by other techniques at a scale below this resolution. Thus, the variance in measurements at each concentration described here could be a result of mimetic tissue model inhomogeneity. This, however, carries over into dosed tissue, where the drug distribution within a single MALDI point could be very heterogeneous. For example, if a certain drug accumulates within a cell organelle, the MALDI result would show only the average drug concentration of the cell. With a diffraction-limited system, such as Raman microscopy used here (spatial resolution ∼1 μm) the drug concentration of the organelle would be much higher within the organelle and reduced elsewhere. This means the limit of detection could still garner useful results even though they are lower than that of less spatially resolved imaging techniques.

The long acquisition times of our instruments generally excludes the application of spontaneous Raman spectroscopy to quantitative imaging or in vivo measurements, as the sample measured must be temporally stable. The high laser powers needed also precludes the use of the technique from live animal studies. However, as the quantification described here was all done using univariate spectral analysis away from the fingerprint region, there is potential for reducing total acquisition time in imaging using wide-field Raman imaging. This is a technique where, instead of a single location being measured across a wide spectral range at a time, a single narrow spectral range is binned and measured in an imaging format. This could essentially allow a whole map to be quantitatively measured in the same time as a single point is using our instruments.^25^ Alternatively, the point-like Raman spectroscopy could be combined with other imaging modalities that can identify different tissue structures and cellular component, such that the Raman spectroscopy could be used for quantification of drug concentration in these different parts of tissue. The large FOV and small FOV mapping shown here demonstrates the capability of Raman spectroscopy for single-point measurement of arbitrary positions within large tissue sections. This could be applied in the context of individual Raman measurements of points smaller than the spatial resolution of other modalities like MALDI-TOF. Similar multimodal spectral imaging where selective-sampling Raman spectroscopy was combined with autofluorescence imaging or real-time compotation of sampling points was previously demonstrated for 10–100-fold decrease in tissue analysis with applications in cancer surgery.^26–29^ The use of software controllable multifoci Raman spectroscopy in power-sharing mode has also been reported as a technique that can be used to measure simultaneous Raman spectra from several locations in tissue.^30,31^

An alternative potential method of quantitative subcellular drug detection is secondary ion mass spectrometry (SIMS) MSI. This is another form of mass spectrometry wherein the ablation and ionization of the sample is performed with an ion beam. This has a spatial resolution < 100 nm,^32^ lower than confocal NIR-based Raman spectroscopy. However, the ionization method in SIMS results in fragmentation of molecules, complicating spectral interpretation.^33^

## Conclusion

Current techniques available for quantifying drugs in tissue (HPLC, mass spectroscopy) are destructive and have poor spatial resolution, limiting cellular and sub-cellular quantitative analysis of tissue. This study shows that spontaneous Raman spectroscopy is a promising technique for quantitative analysis of drug concentration in even highly fluorescent tissues. The linear response of the technique allows simple quantification of measurements, at the cost of longer acquisition times than previously mentioned alternatives. The results show that certain drugs can be measured at pharmacologically relevant concentrations using a diffraction-limited spatially resolved (∼1 μm lateral resolution, ∼10 μm axial resolution), non-destructive technique that requires no sample preparation. This means that Raman spectroscopic measurements can be quantified with only per-instrument calibration, as opposed to per-sample calibration of methods that require sample spraying. A Raman microscope can be calibrated for a given drug/tissue combination using a mimetic tissue model and then used repeatably on multiple dosed tissue samples without additional calibration controls. The thin samples used also enables cross correlation of measurements taken from different analysis modalities, by measuring consecutive sections with different instruments.^34^

While this study demonstrated the potential, further studies are required using intact tissue from animals exposed to medically relevant levels of drugs to evaluate the system on real world dosed tissue. The metabolism of the drugs in dosed animals may sufficiently change the structure of the drugs to the extent that the Raman signature of the drugs is no longer represented by mimetic tissue models.

## Supplemental Material

Supplemental Material - Quantification of Drugs in Brain and Liver Mimetic Tissue Models Using Raman Spectroscopy Click here for additional data file. Supplemental Material for Quantification of Drugs in Brain and Liver Mimetic Tissue Models Using Raman Spectroscopy by Nathan Woodhouse, Jan Majer, Peter Marshall, Steve Hood, and Ioan Notingher in Applied Spectroscopy
