# Supplementary material for: Quantification of Drugs in Brain and Liver Mimetic Tissue Models Using Raman Spectroscopy
Source: Appl Spectrosc. 2022 Nov 16;77(3):246–60. doi: 10.1177/00037028221139494 (PMC10034474; doi:10.1177/00037028221139494)
Supplement: Supplemental Material - Quantification of Drugs in Brain and Liver Mimetic Tissue Models Using Raman Spectroscopy [file sj-pdf-1-asp-10.1177_00037028221139494.pdf]

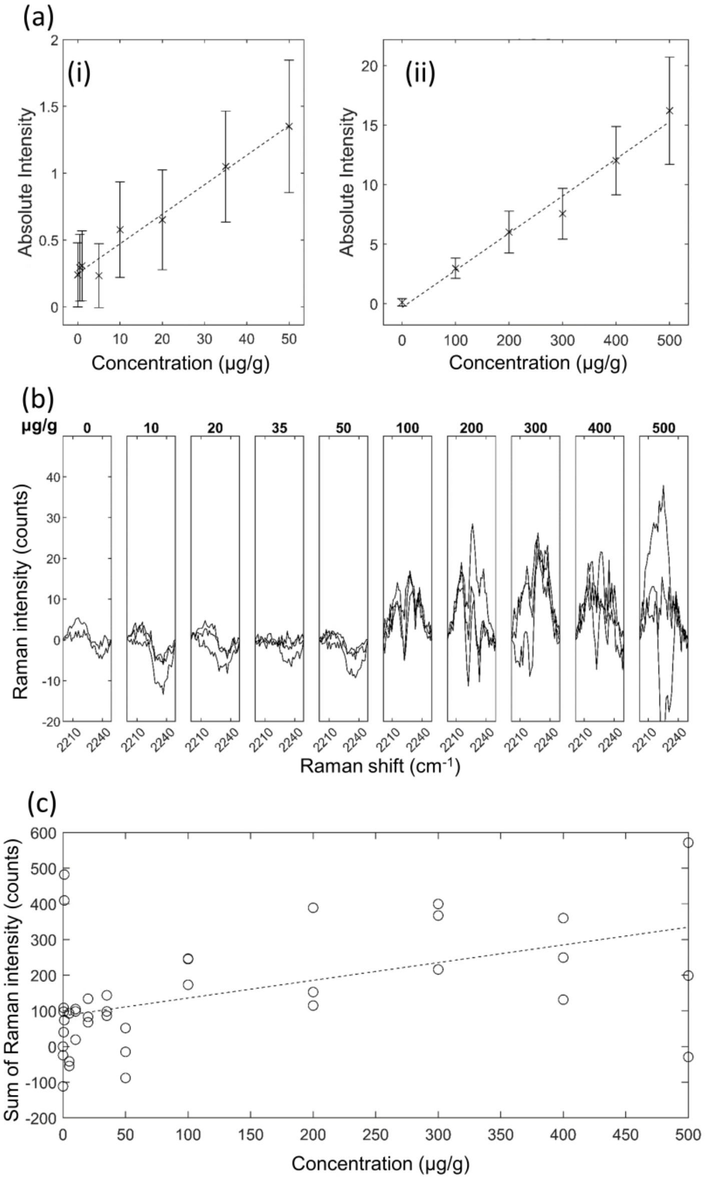

Figure 7. GSK4 in rat liver mimetic tissue model. A(i): Weighted regression fit of MALDI-intensity of 250.6 Da mass peak for 0 – 50 µg/g mimetic tissue model (dotted line), mean measured signal at given concentration and standard deviation (crosses and bars). A(ii): Weighted regression fit of MALDI-intensity of 250.6 Da mass peak for 0 – 500 µg/g mimetic tissue model. B: Integration time-normalized 2236 cm-1 Raman peak in mimetic tissue models (excitation 785 nm). Three randomly sampled locations of each mimetic tissue model were measured for each concentration. C: Linear regression fit of sum of signal under 2236 cm-1 peak at given concentration (dotted line), measured signal under peak at given concentration for each sample (circles).

2066x3404mm (38 x 38 DPI)

SUPPLEMENTAL MATERIAL

Quantification of Drugs in Brain and Liver Mimetic Tissue Models Using Raman Spectroscopy

Nathan Woodhouse[1], Jan Majer[2], Peter Marshall[2], Steve Hood[2], and Ioan Notingher[1]*

[1]School of Physics and Astronomy, University of Nottingham, Nottingham, UK

[2]GlaxoSmithKline, Stevenage, UK

* Corresponding author: ioan.notingher@nottingham.ac.uk

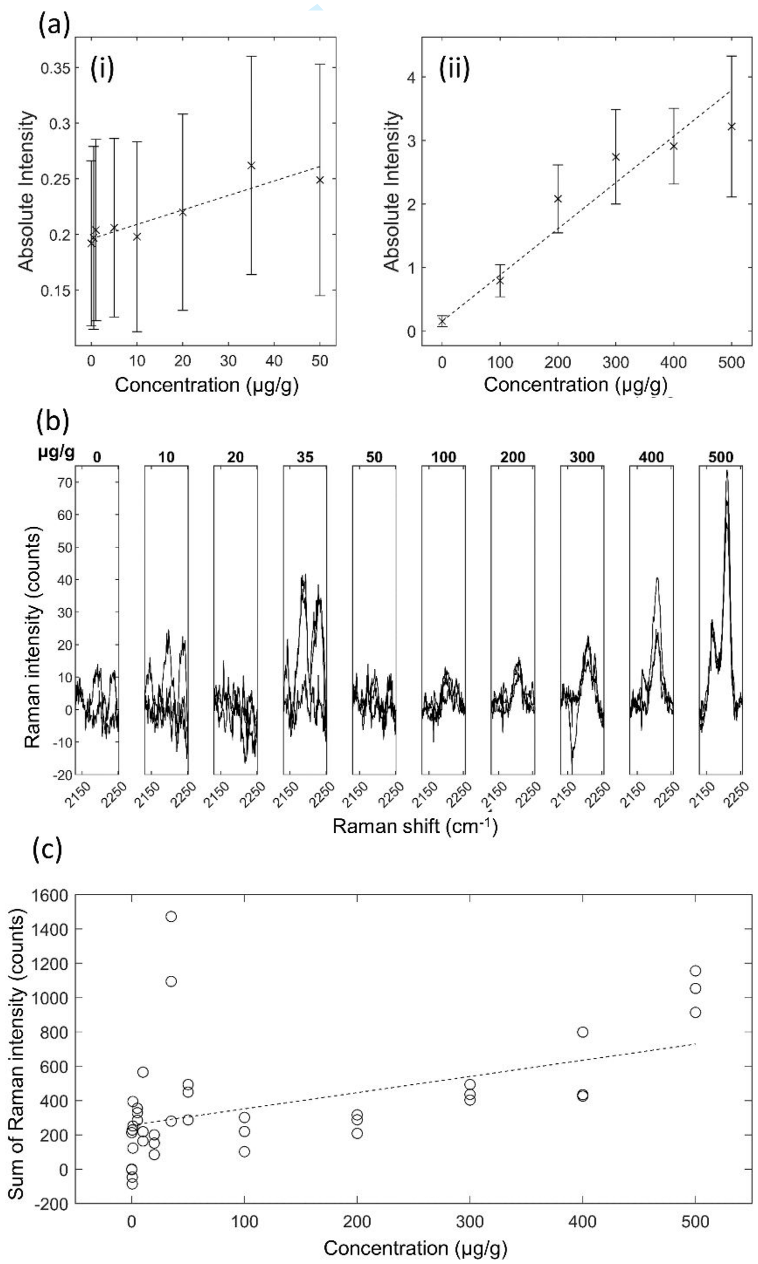

Figure S1. GSK4x in rat brain mimetic tissue model. (a) Weighted regression fit (i) of MALDI-intensity of 242.7 Da mass peak for 0–50 μg/g mimetic tissue model (dotted line),

mean measured signal at given concentration and standard deviation (crosses and bars). (a) Weighted regression fit (ii) of MALDI-intensity of 242.7 Da mass peak for 0–500 µg/g mimetic tissue model. (b) Integration time-normalized 2236 cm^{-1} peak in mimetic tissue models. Three randomly sampled locations of each mimetic tissue model were measured for each concentration. (c) Linear regression fit of sum of signal under 2220 cm^{-1} peak at given concentration (dotted line), measured signal under peak at given concentration for each sample (circles).

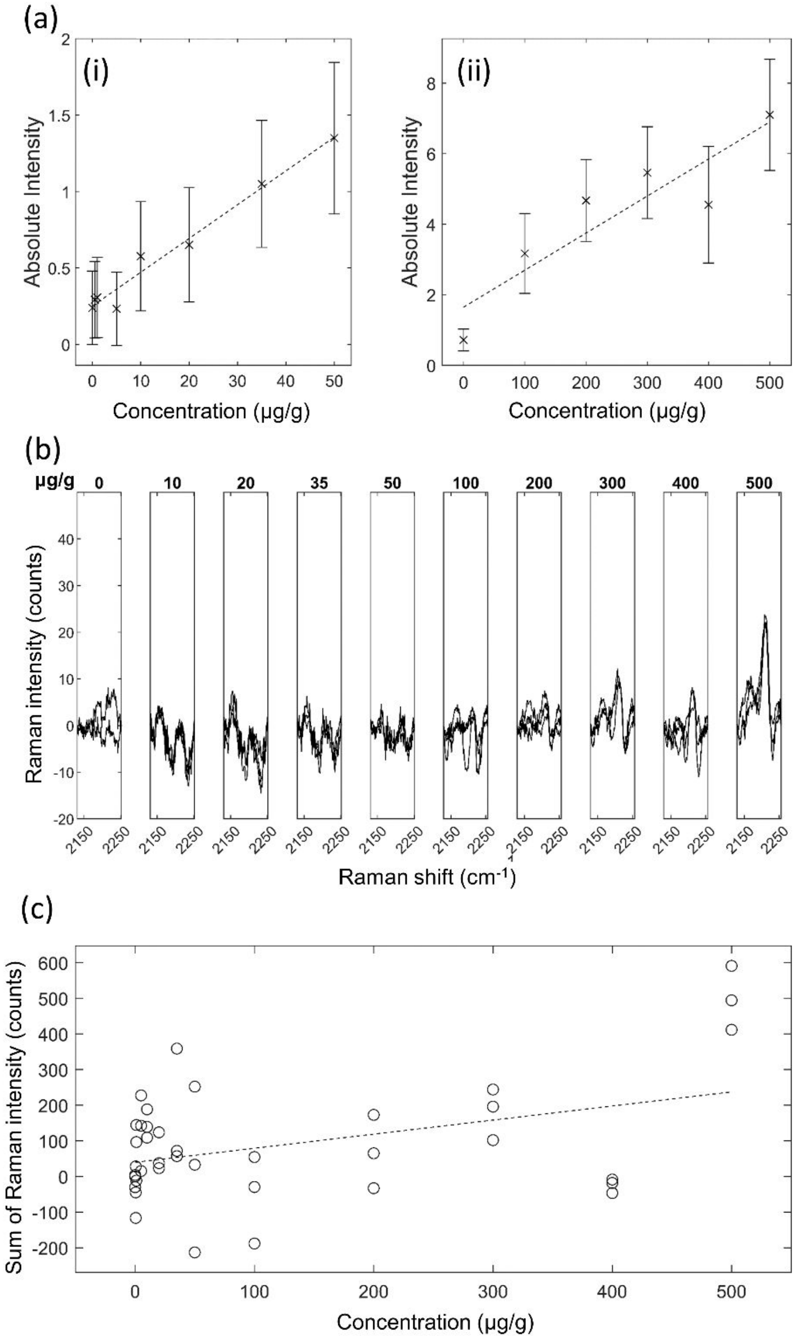

Figure S2. GSK4x in rat liver mimetic tissue model. (a) Weighted regression fit (i) of MALDI-intensity of 242.7 Da mass peak for 0–50 µg/g mimetic tissue model (dotted line),
